# Potential Prognostic Markers of Acute Kidney Injury in the Early Phase of Acute Pancreatitis

**DOI:** 10.3390/ijms20153714

**Published:** 2019-07-30

**Authors:** Justyna Wajda, Paulina Dumnicka, Małgorzata Maraj, Piotr Ceranowicz, Marek Kuźniewski, Beata Kuśnierz-Cabala

**Affiliations:** 1 Department of Anatomy, Faculty of Medicine, Jagiellonian University Medical College, 31-034 Kraków, Poland; 2 Department of Medical Diagnostics, Faculty of Pharmacy, Jagiellonian University Medical College, 30-688 Kraków, Poland; 3 Department of Physiology, Faculty of Medicine, Jagiellonian University Medical College, 31-531 Kraków, Poland; 4 Chair and Department of Nephrology, Faculty of Medicine, Jagiellonian University Medical College, 31-501 Kraków, Poland; 5 Department of Diagnostics, Chair of Clinical Biochemistry, Faculty of Medicine, Jagiellonian University Medical College, 31-501 Kraków, Poland

**Keywords:** acute kidney injury, acute pancreatitis, biomarkers, inflammation, diagnostic accuracy

## Abstract

Acute kidney injury (AKI) is a serious complication of acute pancreatitis (AP), which occurs in up to 70% of patients with severe AP and significantly increases the risk of mortality. At present, AKI is diagnosed based on dynamic increase in serum creatinine and decreased urine output; however, there is a need for earlier and more accurate biomarkers. The aim of the study was to review current evidence on the laboratory tests that were studied as the potential biomarkers of AKI in AP. We also briefly summarized the knowledge coming from the studies including sepsis or ICU patients since severe acute pancreatitis is associated with systemic inflammation and organ failure. Serum cystatin C and serum or urine NGAL have been shown to predict or diagnose AKI in AP; however, this evidence come from the single center studies of low number of patients. Other markers, such as urinary kidney injury molecule-1, cell cycle arrest biomarkers (tissue inhibitor metalloproteinase-2 and urine insulin-like growth factor-binding protein 7), interleukin-18, liver-type fatty acid-binding protein, or calprotectin have been studied in other populations suffering from systemic inflammatory states. In AP, the potential markers of AKI may be significantly influenced by either dehydration or inflammation, and the impact of these factors may be difficult to distinguish from kidney injury. The subject of AKI complicating AP is understudied. More studies are needed, for both exploratory (to choose the best markers) and clinical (to evaluate the diagnostic accuracy of the chosen markers in real clinical settings).

## 1. Introduction

Acute kidney injury (AKI) is a rapid decline of renal excretory function that may be caused by various etiological factors. Its mechanism can vary, as it can develop after major surgeries, sepsis, in consequence of low cardiac output, hypovolemia, rhabdomyolysis, urinary obstruction, or drug toxicity [1]. The symptoms of the underlaying disease are accompanied by the symptoms of inadequate elimination of nitrogenous waste products, such as weakness, nausea, vomiting, or loss of appetite [1]. The urine output often decreases, but it may also be within the normal range or even increased. The results of laboratory tests: elevated serum urea and creatinine concentrations reflect the diminished elimination of nitrogen waste products.

It is estimated that from about 7% to more than 20% of hospitalized patients develop AKI [1,2]. In a group of critically ill patients, the incidence increases to as much as 50%, and it is tightly linked to mortality [1,3]. The American data reveal an increasing number of patients who develop dialysis-requiring AKI and an increasing number of deaths from AKI [4]. Recent meta-analysis estimates that the overall mortality in AKI exceeds 20% [2].

The critically ill patients that require treatment in intensive care unit (ICU) constitute the population that is most often studied in the context of AKI. However, ICU patients suffer from the most severe conditions, have multiple comorbidities, and are most aggressively treated, thus, the conclusions of such studies cannot be easily translated to other populations [4]. AKI puts additional strain on a critically ill patient, which significantly increases mortality risk [5]. The mechanism of renal failure in critically ill patients is complex, with overlapping factors. These include hemodynamic changes and microcirculatory dysfunction, constriction of renal vessels, increase in intraabdominal pressure, the injury related to systemic inflammation, and the influence of nephrotoxic drugs. Adverse clinical outcome can be further influenced by factors, such as male gender, elderly age, sepsis, lung or liver failure, comorbidities, and other severe conditions [6].

Acute pancreatitis AP represents an inflammatory disorder that is associated with variable severity. A major event in the pathophysiology of the disease is the premature activation of pancreatic enzymes within the gland associated with injury to acinar cells and intrapancreatic inflammatory response [7]. Various causes may lead to AP; however, patients with biliary stones or sludge and those consuming excess of alcohol represent the majority of AP cases. Pancreatic duct obstruction and toxic effects of bile acids and alcohol both have been pathophysiologically linked to the premature activation of trypsinogen to trypsin, the most powerful pancreatic protease (reviewed in [8]). Rarer causes include hypertriglyceridemia, post-endoscopic retrograde cholangiopancreatography complication, autoimmune diseases, trauma, infections, adverse drug reactions, or genetic disorders (mutations of *PRSS1, SPINK1,* or *CFTR* genes) [7]. Local pancreatitis may resolve without complications; however, excessive pancreatic injury and inflammation is associated with pancreatic necrosis, release of pancreatic enzymes, inflammatory cytokines and damage-associated molecular patterns into systemic circulation, systemic activation of inflammatory cells, kinin pathway and complement, endothelial dysfunction, coagulation abnormalities, and oxidative stress [7,8]. AP is diagnosed based on characteristic acute abdominal pain (often accompanied with nausea and vomiting), any abnormal results of laboratory tests for pancreatic enzymes (serum lipase or amylase at least three times above the upper reference limit), and the signs that were observed in abdominal imaging (contrast-enhanced computed tomography, magnetic resonance imaging, or ultrasonography) [9]. Most commonly used consensus, the Atlanta classification revised in 2012 [9] defines three grades of AP severity: mild AP not associated with local or systemic complications; moderately-severe AP associated with either local complications, exacerbation of preexisting conditions, or short-lasting (resolving within 48 h) organ failure; and, severe AP associated with persistent (i.e., lasting longer than 48 h) organ failure.

AKI is a well-known complication of AP, regarding about 20% of patients, although the prevalence rates varies across the studies (Table 1). A multicenter study that was conducted by Zhou et al. [10] has shown that AKI occurs in almost 70% of cases of severe acute pancreatitis (SAP) that were admitted to ICU. Of note, almost half of AKI cases in this study were classified as stage 3 according to Kidney Disease: Improving Global Outcomes (KDIGO) criteria [10]. In a retrospective analysis of over 220,000 ICU admissions, Lin et al. [11] observed AKI that among patients with AP was more common (with odds ratio of 4.862) than in patients with other diagnoses. The prevalence of AKI in ICU patients with AP (15.05%) even exceeded the prevalence of AKI in sepsis (13.2%). Additionally, SAP was one of the eight most common surgical diagnoses among the patients with AKI [12]. The development of AKI in patients with SAP significantly increases the risk of death: among the patients with SAP, the mortality rates are doubled in those who developed AKI (over 40% versus 20%) [10,13] (Table 1). On the other hand, a recent study showed that isolated renal failure in SAP is associated with better prognosis and less mortality than multi-organ failure and it requires much shorter ICU admissions than isolated respiratory failure (mean length of ICU stay of 2.4 and 15.7 days, respectively) [14]. 

**Figure 1:**
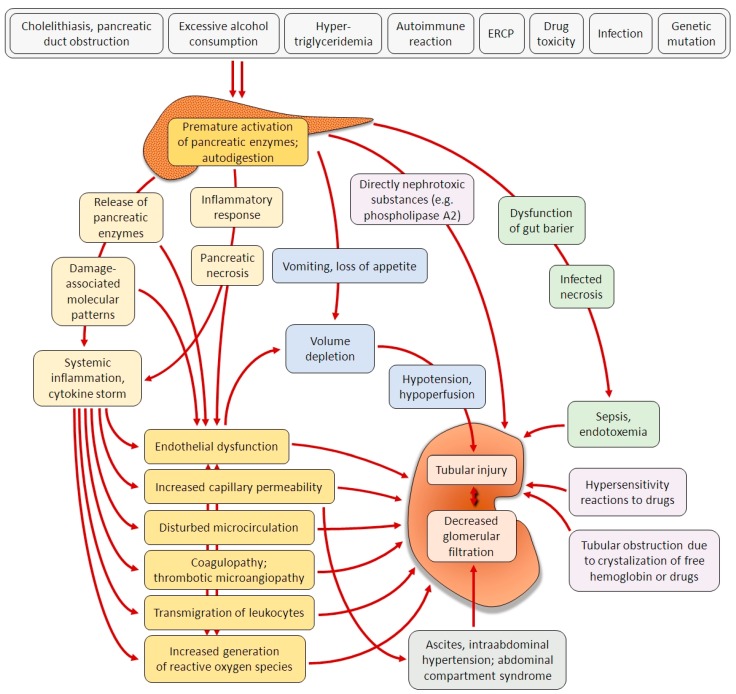
Pathophysiological factors influencing the development of AKI in AP.

Pathophysiological mechanisms that are responsible for kidney failure in the course of AP are diverse and multifactorial (Figure 1). The clinical symptoms associated with AP, such as vomiting and the loss of appetite result in fluid depletion. In the course of AP, toxins, free radicals, cytokines, and other inflammatory mediators are released to the circulation, which lead to endothelial dysfunction and increased permeability of blood vessels [8], which further exacerbates the hypovolemia. The systemic inflammatory response causes the constriction of blood vessels and the stimulation of baroreceptors. The redistribution of body fluids and the shift towards the third space decrease the intravascular volume. These mechanisms lead to hypoperfusion of the kidneys. Additionally, the intrabdominal pressure increases due to ascites and sometimes hemorrhages, leading to the development of abdominal compartment syndrome and the decline in renal perfusion. Ischemia and oxygen deficiency lead to the impairment of renal function [16,19]. Higher fluid sequestration in AP has been associated with more severe course of the disease being reflected by more organ failure [20]. However, the disrupted control of microvascular pressure tone that is observed in consequence of systemic inflammation may be a more important factor leading to kidney injury. An aggressive fluid resuscitation in AP has been associated with more renal failure in recent clinical studies [21,22]. The aggressive fluid resuscitation efficiently reverses renal hypoperfusion; however, it cannot reverse the microcirculation failure. The other factors that are associated with kidney injury in AP may include thrombotic microangiopathies [23], adverse reactions to drugs [24], hypertriglyceridemia [25,26], or the apoptotic cell death of renal tubules and necrosis that are caused by the accumulation of phospholipase 2 released in the course of AP [27]. Recent animal experiment demonstrated the potential of antithrombin III to alleviate renal injury in AP [28]. In a later stage of AP, bacterial infection and endotoxemia become the important factors in the development of organ failure [29]. Table 2 summarizes recent pathophysiological data stemming from animal studies on AKI as a complication of AP.

At present, the diagnosis of AKI is based on the dynamic increase in serum creatinine and/or decreased urine output [30]. However, a substantial increase in serum creatinine is regarded as a late sign of AKI. There has been a continuous search for an adequate biomarker, which could outperform creatinine, allowing for early diagnosis and treatment of AKI before the irreversible changes occur. An ideal biomarker of AKI should allow for early detection with high diagnostic sensitivity and specificity. It should remain detectable long enough to allow for diagnosis in real clinical settings, but change quickly enough to allow for monitoring of the course of the disease. Additionally, a diagnostic test used for the detection of AKI should be minimally invasive, widely available, cheap, easy to conduct, and reproducible. An ideal biomarker should predict the course of the disease and its outcome.

Our aim was to review current evidence on the laboratory tests studied as the potential biomarkers of AKI in AP. However, renal failure accompanying AP is understudied. Simultaneously, there is growing evidence on laboratory markers supporting the diagnosis of AKI in patients with sepsis and other acute states that require intensive care. We briefly summarized the knowledge coming from the studies, including sepsis or ICU patients to show the data on AKI complicating AP in a broader context, since severe acute pancreatitis is associated with systemic inflammation and organ failure.

## 2. The Markers of Glomerular Filtration

### 2.1. Serum Creatinine

Creatinine is a parameter that is widely used in the clinical practice; nevertheless, it has significant limitations. Creatinine is mainly filtered in renal glomeruli, but it is also secreted in renal tubules, a process that can be influenced by non-renal factors, such as medication [31]. Additionally, the concentrations of creatinine in serum depend on age, gender, muscle mass, and hydration status [32]. Sudden changes in glomerular filtration do not always correlate with creatinine, because of its relatively long half-life. The accurate estimation of glomerular filtration rate based on serum creatinine requires the steady state between creatinine production and excretion—these conditions are not fulfilled in AKI [31]. A substantial increase in serum creatinine occurs after the 50% drop in the glomerular filtration rate [33]. Therefore, especially in early stages of kidney injury, creatinine concentration in serum does not accurately reflect disease severity, and its interpretation can be misleading [32,33].

### 2.2. Serum Cystatin C

The evidence regarding the usefulness of serum cystatin C in the course of AKI is inconsistent. Cystatin C is a low molecular weight protein that is produced by all nucleated cells of the body, filtered through glomeruli, and almost completely reabsorbed in the proximal tubule [33]. Serum (or plasma) concentrations of cystatin C is rather independent of factors, such as age and gender, but hypo- or hyperthyroidism, insulin, or glucocorticoid administration must be considered [33]. Cystatin C is documented to be a good marker of AKI in sepsis, allowing for prognosis 24 h before diagnosis [33,34]. In a study of 113 patients that were admitted to ICU with sepsis [33], cystatin C concentration was evaluated on days following admission and then compared with routinely assessed serum creatinine concentration. Both serum creatinine and cystatin C were increased in patients with AKI at all study days [33]. The measurement of cystatin C concentration in the serum was also proposed for monitoring renal replacement therapy [35]. In 2018, Chai et al. [13] assessed the diagnostic accuracy of serum cystatin C among 237 patients with AP, of who 18 developed AKI. At a cut-off of 1.865 mg/L, the diagnostic sensitivity was estimated for almost 90% and specificity for 100%; the area under the receiver operating characteristic (ROC) curve was 0.948 [13] (Table 3).

## 3. The Markers of Tubular Dysfunction

### 3.1. Neutrophil Gelatinase-Associated Lipocalin (NGAL)

NGAL is a glycoprotein that is expressed in many cells of the body i.e., hepatocytes, kidney tubular cells, and endothelial cells, but it was initially isolated from neutrophils’ granules [40]. NGAL in humans is present in blood and urine in three different forms: a monomer, homodimer, and heterodimer (covalently bound to gelatinase, or matrix metalloproteinase 9). Endothelial cells of kidney tubules produce monomers (ascending part of the loop of Henle and collecting duct), and dimers by neutrophils, which can also secrete monomers [41].

NGAL is actively synthesized in the distal part of a nephron following tubular injury. Moreover, NGAL (present in plasma) is filtered through the glomeruli and is reabsorbed in the proximal part of the convoluted tubule; however, the reabsorption is diminished in case of proximal tubule dysfunction [42,43,44]. These two mechanisms may be responsible for increased urinary concentrations of NGAL in AKI. Simultaneously, the plasma NGAL concentrations increase. Urine NGAL concentrations rise quickly in AKI and dynamically change, which allows for the monitoring of kidney injury [45,46]. The NGAL measurements can have been shown to predict AKI that is caused by bacterial infection [47] and sepsis [34]. A meta-analysis on the diagnostic accuracy of plasma NGAL measurements to diagnose AKI among patients with sepsis [48] has shown high diagnostic sensitivity (88.1%) but low specificity (47.4%). Another meta-analysis summarized the evidence regarding the diagnosis of AKI among patients of critical care units [49]. The diagnostic sensitivity and specificity of plasma NGAL in this setting have been estimated for 72% an 81%, while the diagnostic sensitivity and specificity of urine NGAL have been estimated for 70% and 79%, respectively [49].

Siddappa et al. [50] evaluated serum and urine NGAL as the prognostic markers of AKI in a small cohort of patients with AP and reported satisfying diagnostic accuracy of the markers that were measured on day 1, following patients’ admission (area under the ROC curve of 0.8 and 0.9, respectively) (Table 3). In another study of 65 patients with AP, the concentrations of NGAL measured in serum and in urine on admission and two subsequent days were significantly higher in 11 patients who developed AKI in comparison to those who did not [51]. Additionally, the concentration of serum [52] and urine [53] NGAL correlated with AP severity that was evaluated according to Atlanta classification, BISAP scale, Marshall scale, imaging test results, and selected markers of inflammation (Table 4). However, these studies [52,53] did not report the association of serum or urine NGAL with AKI complicating AP or the proportion of AP patients who developed AKI. Serum NGAL, as measured by Sporek et al. [52] at 24, 48, and 72 h from the onset of AP, increased between the first and second day of AP, which was reflected by higher cut-off value to differentiate between mild and moderately severe/severe AP at 48 h (183 μg/L versus 165 μg/L at 24 h) (Table 4).

### 3.2. Kidney Injury Molecule 1 (KIM-1)

KIM-1 is a transmembrane glycoprotein that is expressed on epithelial cells of renal proximal tubule after ischemia or toxic injury [40]. Extracellular domain of KIM-1 is cleaved and released to urine under the influence of kidney damaging factors [54]. The KIM-1 concentrations in urine are positively correlated with the degree of renal damage, but they also indicate the severity of inflammation. [32] The presence of KIM-1 in urine is a sign of kidney (proximal tubule) damage, since in healthy kidneys KIM-1 is present in very low (or undetectable) concentrations [1]. KIM-1 seems to be a valuable marker of AKI when considering its very low concentrations in healthy kidneys, the noticeable response to renal ischemia with reperfusion and the expression on tubular cells until renal recovery [55,56]. Its urine concentrations increase in the course of AKI in comparison to those with chronic kidney disease without AKI [57] and have been suggested to predict renal outcomes following AKI [58].

A study of patients with sepsis has reported a high area under curve value (AUC 0.912) for KIM-1 in diagnosis of AKI at the 24 h following ICU admission [59]. A significant increase in urinary KIM-1 was confirmed as soon as after 6 h from ICU admission and it remained elevated for 48 h. Additionally, a higher concentration of KIM-1 was observed in the patients who died [59]. In turn, metanalysis gathering 11 clinical studies has shown that diagnostic sensitivity for KIM-1 in AKI prognosis is 74% and specificity reaches 86% [60]. Urinary KIM-1 has been shown to predict of the need for dialysis and the risk of death in patients with AKI [61,62,63]. To our best knowledge, no studies have been published regarding the diagnostic performance of KIM-1 in renal failure that is associated with AP.

### 3.3. Tissue Inhibitor Metalloproteinase-2 (TIMP-2) and Urine Insulin-Like Growth Factor-Binding Protein 7 (IGFBP7)

In 2013, Kashani et al. [68] published the results of a large study, including ICU patients, introducing TIMP-2 and IGFBP7 as the early markers of AKI. The diagnostic accuracy of the combination of the two proteins measured in urine has been shown to outperform numerous other potential biomarkers [68]. The expression of TIMP-2 and IGFBP-7 increases in the renal tubular cells (of distal and proximal tubule, respectively) in the early phase of cellular injury and their increase induces cell cycle arrest in G1 phase [69]. In the experimental study on rats, TIMP-2 and IGFBP7 in combination had better diagnostic accuracy for AKI that each marker alone [70]. Similarly, in the American multi-centered study on 232 patients with sepsis, the area under ROC curve was 0.84 for the markers used in combination [71]. Metanalysis that was conducted by Liu et al. [69] covering nine studies that were conducted throughout 2013–2016, encompassing 1886 adult patients from America and Europe, has proved the usefulness of combined TIMP-2 and IGFBP7 as reliable AKI markers, with the combined area under the ROC curve of 0.86. Another meta-analysis encompassing 10 prospective studies described by Su et al. [72] has shown the diagnostic sensitivity of 0.84, specificity of 0.57, and area under ROC curve of 0.88. However, both meta-analyses [68,72] were based on heterogenous studies, including patients in various health-care settings (mainly critically ill ICU patients and post-surgical patients). Some limitations of the TIMP-2 and IGFBP-7 have been suggested, such as dependence on age [73] or increase in diabetic patients [74]. There are no studies evaluating this promising combination of markers in patients with AP. Additionally, we have not found the studies evaluating IGFBP-7 in AP in any way (neither human nor animal). 

TIMP-2, as an inhibitor of extracellular matrix metalloproteinases (MMPs), was mostly studied in the context of chronic pancreatitis, where MMPs are implicated in extracellular matrix remodeling and pancreatic fibrosis [75,76]. Matrix metalloproteinase-9 (MMP-9) was implicated in renal injury in SAP: MMP-9 seems to be involved in renal capillary injury and leakage, as its expression in kidneys preceded the histological signs of renal injury [37] (see Table 2). Additionally, earlier works have shown the pathophysiological role of MMP-9, being released by activated neutrophils, in gut-barrier disruption and organ failure in SAP [77,78]. Nonetheless, to our knowledge, there are no reports on TIMP-2 in association with AP, or AKI in AP, although it seems to be reasonable to study the marker in this context. In a small study of Wereszczyńska-Siemiątkowska et al. [79], TIMP-1 (a more potent inhibitor of MMP-9 than TIMP-2), although it was increased in patients with (mild and severe) AP in comparison.to healthy controls, was shown to be too low to inhibit the increased MMP-9 levels. More recently, Nukarinen et al. [80] have shown the high diagnostic accuracy of MMP-8 in differentiation between mild and moderately-severe AP and SAP (area under the ROC curve of 0.939). MMP-7 did not differ according to AP severity, MMP-9 and TIMP-1 only differed significantly between mild and severe AP; moreover, MMP-7 and TIMP-1 significantly correlated with serum creatinine in 176 patients with AP [80]. The study [80] confirmed the higher concentrations of MMP-7 to −9 and TIMP-1 among patients with AP in comparison to the healthy controls.

### 3.4. Interleukin 18 (IL-18)

Interleukin-18 is a proinflammatory cytokine that is released by monocytes/macrophages and other antigen presenting cells, which belongs to interleukin-1 family, and its conversion to an active form is mediated by caspase-1 [81]. IL-18 is an inflammatory mediator that is produced in response to ischemia of various organs i.e., kidneys, heart, or brain. Its concentrations in serum increase in sepsis, joints inflammation, liver inflammation, inflammatory bowel syndrome, and lupus [1]. It has been observed that increase in IL-18 urine concentrations occurs relatively fast in response to renal tubular injury [81]. In consequence of kidney damage, an increased concentration in urine is observed in the first 6 h with the peak between 12 and 18 h [56]. The animal studies imply the pathological role of IL-18 in the development of tubular damage in AP. Significant morphological changes have been observed as soon as after 12 h from AP onset in the microscopic evaluation of kidneys of rats with SAP [36]. In ischemia-reperfusion induced kidney injury in mice, IL-18 has been documented to be a key component in the development of AKI [82]. Lin et al. [83] conducted a meta-analysis that evaluated urinary IL-18 as a marker of AKI, summarizing 11 very heterogenous studies, including patients in any age (also neonates), treated in ICU, or following cardiac surgery, implementing various definitions of AKI. The pooled area under the ROC curve was AUC 0.77 [83]. The diagnostic performance of IL-18 was better in children than in adults [83]. No clinical studies regarding AKI in AP have been published. However, in the light of the role of IL-18 in AKI pathomechanism that was suggested by the results of experimental studies, further research on this marker seems justified [81].

### 3.5. Liver-Type Fatty Acid-Binding Protein (L-FABP)

L-FABP is a low molecular weight protein that is mainly produced in the liver, but is isolated from other organs i.e., kidneys, stomach, intestines, and lungs. The main function of the family of compounds comprising L-FABP is the binding of fatty acids and their transmembrane transport. L-FABP is filtered in renal glomeruli and then reabsorbed in the proximal tubule. In a healthy individual, its concentration in urine is very low. Its expression is induced by hypoxia and has been proposed as a marker of kidney function in patients post kidney transplantation, where its concentrations correlate with the duration of ischemia [84]. Other insults may also induce an increased secretion of L-FABP to the lumen of proximal tubule and the elevated concentration in urine, proportional to the severity of kidney damage [57]. The L-FABP urine concentrations have been shown to increase in sepsis, contrast-induced kidney injury, heart failure, or after cardiac surgery [1].

In a study that was conducted on 145 patients with AKI in the course of septic shock, higher concentrations of L-FABP in urine were observed in patients who died and it prognosed mortality with high accuracy (the area under the ROC curve of 0.99) [85]. In another study on 85 patients, high diagnostic usefulness of urine L-FABP has been observed in the early prognosis of AKI after cardiac surgery [86]. In the meta-analysis of the studies on AKI post cardiac surgery [87], urine L-FABP was a moderately good prognostic marker of AKI (area under the ROC curve of 0.72). Additionally, in patients of ICU, higher urine L-FABP at admission has been associated with a higher risk of AKI [88]. Higher urine L-FABP has been shown to predict disease progression, need for dialysis and death among 152 ICU patients in the early phase of AKI [89]. The marker has not been evaluated in the prediction or diagnosis of AKI complicating AP.

### 3.6. Calprotectin

Calprotectin is an established marker of local inflammation. It is a protein consisting of two units: S100A8 and S100A9 and neutrophils and renal tubular cells produce it [90]. In several studies, calprotectin has been recognized as a marker that differentiates between the kidney injury of pre-renal and intra-renal origin. Heller et al. [91] and Seibert et al. [92] reported the usefulness of calprotectin measurements in urine in the detection of AKI. The diagnostic sensitivity and specificity were high (over 90%), although the cut-off values differed between the studies from 219.8 ng/mL [93] to 440 ng/mL [92]. The usefulness of urine calprotectin has also been reported by Chang et al. [94], who studied patients admitted to coronary care unit. They reported high diagnostic accuracy (area under the ROC curve of 0.946) for the diagnosis of AKI [94]. Gao et al. [95] and Lee et al. [93] observed higher urine calprotectin in patients with sepsis complicated with AKI (with areas under the ROC curves of 0.901 and 0.889, respectively). Again, there are no studies in AP patients.

### 3.7. Urinary β2-Microglobulin

As a low molecular weight protein, serum β2-microglobulin has been suggested as a marker of glomerular filtration glomerular injury, although the serum concentrations also increase in non-renal diseases such as leukemia, lupus and Crohn’s disease [96]. In the urine of a healthy human the concentrations of β2-microglobulin are very low [96]. However, the increased concentrations in urine may reflect the injury of proximal tubule that was associated with diminished reabsorption of the protein [97]. In the study on 252 children admitted to emergency center, β2-microglobulin in urine was effective in detecting AKI [98]. Two small studies assessed urinary β2-microglobulin in patients with AP [99,100]. One [99] did not found differences in urine β2-microglobulin concentrations between the patients with mild and severe AP. The newer one [100] assessed urinary β2-microglobulin to saponin ratio while using mass spectrometry technology and found increased ratios in severe AP correlated with kidney injury.

### 3.8. Monocyte Chemoattractant Protein (MCP-1)

Monocyte Chemoattractant Protein (MCP-1) is a pro-inflammatory chemokine, which takes part in the recruitment of monocytes during infection or injury. It is secreted in proximal tubules in response to ischemia [101,102]. MCP-1 plays a major role in selectively recruiting monocytes, neutrophils, and lymphocytes in response to proinflammatory cytokines [103]. Increased plasma concentrations of MCP-1 can predict AKI or death in patients after cardiac surgeries [104]. Serum or plasma MCP-1 have been shown to increase in severe AP as a marker of inflammation [105,106,107].

### 3.9. Uromodulin

Uromodulin (or Tamm-Horsfall protein) is a glycoprotein that is exclusively produced by renal tubular cells of the thick ascending part of the loop of Henle [65]. Recently, serum uromodulin concentrations have been shown to reflect the mass of remaining renal tissue and strongly positively correlate with GFR values across all stages of chronic kidney disease [108]. In early phase of AP, serum uromodulin positively correlated with eGFR (independently of sex and age) and negatively with serum creatinine and cystatin C; however, the correlations were much weaker as compared to what has been observed in chronic kidney disease [65]. However, the diagnostic accuracy of serum uromodulin for the diagnosis of AKI complicating the early phase of AP (area under the ROC curve of 0.684) was lower than serum creatinine or cystatin C (Table 3) [65]. 

## 4. Other Markers Associated with Kidney Injury in AP

The markers of endothelial dysfunction: angiopoietin-2 and serum fms-like tyrosine kinase-1 (sFlt-1) have been shown to correlate with serum creatinine, serum cystatin C, serum, and urine NGAL in the early phase of AP [66,67,109]. In a small single-center study, serum angiopoietin-2 has been shown to positively predict AKI diagnosed, according to KDIGO and renal failure diagnosed according to modified Marshall scoring system during first 72 h of AP [66]. Additionally, sFlt-1 significantly predicted renal failure in the first two days of AP [67] (Table 3). Increased expression of a protein that is associated with remodeling of endothelial cells’ cytoskeleton and junctions, vasodilator-stimulated phosphoprotein (VASP) was observed in kidneys of rats with AP [37]. VASP expression seemed involved in pathophysiology of AKI complicating AP, as its maximum preceded the maximum histological changes that are associated with renal failure [37] (Table 2).

Procalcitonin is a polypeptide precursor of a hormone calcitonin. In thecase of bacterial infections, it is extensively produced by many cells, including monocytes and macrophages [110]. Huang et al. [64] demonstrated that higher serum procalcitonin predicted AKI among patients with AP with high diagnostic accuracy (Table 3). Other inflammatory markers are also associated with AKI in AP. During the first two days of AP, serum urokinase-type plasminogen activator receptor (uPAR) positively predicted AKI [111], while serum interleukin 6 positively correlated with renal markers (cystatin C and NGAL) [112]. 

## 5. Associations between Renal Markers and AP Severity

On the other hand, fluid depletion and sequestration in AP are known prognostic factors of a severe course of the disease [20]. Additionally, the increased intra-abdominal pressure predicts severe course of AP [113]. Simultaneously, these factors are involved in pathomechanism of kidney injury [29]. Consequently, the markers of renal function and injury have been associated with severity of AP. Blood urea nitrogen has been included in Ranson and Glasgow/Imrie scores, as one of the variables significantly associated with SAP [7]. This has been confirmed in more recent analyses and BUN is also a part of BISAP and BALI models that were used in early prediction of SAP [114,115]. Additionally, serum and urine NGAL have been reported to predict SAP (Table 4).

## 6. Conclusions

AKI has long been recognized as a complication of severe AP, which is associated with increased mortality. However, the good quality studies on this condition are insufficient. Even the recent epidemiological studies (Table 1) reported conflicting results, with the prevalence of AKI in SAP ranging from 15% [11] to 70% [10]. Several issues may be related to this discrepancy. Although we have concentrated on data based on the 2012 revision of Atlanta classification [9], the real clinical severity of patients that were admitted to various centers might have, in fact, varied depending on the local settlements, e.g., the availability of ICU care may differ between the centers. The studies analyzing only the most severe cases admitted to ICU tend to report higher prevalence of AKI [10]. Fluid resuscitation is of utmost importance in the early phase of AP; however, the controversies regarding the extent of hydration are they still not fully resolved and there may be differences between centers; both too less and too much fluid seems deleterious to the kidneys [21,22,116]. Probably, the most important discrepancies between the epidemiological studies result from various definitions of AKI used: although KDIGO consensus is available since 2012 [117], the AKIN and RIFLE criteria were also used in the reviewed studies; moreover, some studies do not report anuria/oliguria, only relying of serum creatinine (e.g., [10]). In AP, the modified Marshall scoring system (as suggested by 2012 Atlanta classification [9]) is also used to define “renal failure”, with a cut-off value of serum creatinine over 170 µmol/L, giving very different epidemiological data as compared to KDIGO AKI. 

Except for the most extensively renal markers reviewed above, other potential markers, such as urine calbindin, glutathione transferase, clasterin, osteopontin, or trefoil factor-3, have been proposed in the prediction or early diagnosis of AKI in various clinical settings [1,118,119,120,121]. However, there are no data regarding these markers among patients with AP. In general, the studies on biomarkers of AKI complicating AP are insufficient, and the existing clinical data are based on the observation of a limited number of patients (Table 3). Moreover, the potential markers of AKI may be significantly influenced in patients with (severe) AP by either dehydration or inflammation, and the impact of such factors may be difficult to distinguish from kidney injury itself; this seems to be a significant issue e.g., with inflammatory markers (procalcitonin, uPAR, IL-6). However, from existing evidence on the markers of AKI in AP, the diagnostic usefulness of procalcitonin was slightly better than that reported for cystatin C and NGAL (Table 3). Among the most recognized kidney injury markers, serum cystatin C (the marker of glomerular filtration) and serum or urine NGAL (the markers of tubular injury) have also been reported to predict or diagnose AKI in AP with good diagnostic accuracy; however, even this evidence come from the single center studies of low number of patients. The advantages of serum procalcitonin, serum cystatin C, and urine NGAL include the fast and robust fully automated laboratory methods, allowing for implementing their measurements in practically every routine medical laboratory. This should encourage larger clinical studies on these markers in near future, and such studies are necessary to verify the available single-center data.

The other markers already well studied in ICU patients or sepsis (e.g., KIM-1, IL-18, IGFBP-7, and TIMP-2) should be verified in the AP patients. However, we should also look for serum or plasma markers, which would be measurable in patients with significant oliguria or anuria at the early phase of AP. There are also interesting emerging markers, e.g., VASP, that need to be better characterized. More studies are needed regarding AKI complicating AP, both exploratory (to choose the best markers, or probably a combination of markers, a strategy that worked well in the case of IGFBP-7*TIMP-2) and clinical (to evaluate the diagnostic accuracy of the chosen markers in real clinical settings). Happily, in recent years, the subject of AKI complicating AP seems to gain more interest, as reflected by more published reports.

## Figures and Tables

**Table 1:** The data on the prevalence of acute kidney injury (AKI) in patients with acute pancreatitis (AP).

Reference	Study Design and Patients	Definition of AKI	Prevalence of AKI	Remarks
Lin et al., 2011 [11]	Retrospective study of 1734 patients with AP admitted to ICU	AKI was identified using the ICD-9 code 584 (AKI).	15.05% of ICU patients with AP	Mortality was 23.76% in AP with AKI versus 8.08% in AP without AKI.
Pavlidis et al., 2013 [15]	Retrospective analysis of 50 patients with SAP admitted to ICU	AKI was defined according to AKIN criteria.	54% of patients with SAP; 44% of patients with SAP required RRT	AKI more common among non-survivors (100%) than survivors (42%)
Zhou et al., 2015 [10]	Retrospective multi-center analysis of 414 patients with SAP admitted to ICU	AKI was defined according to AKIN criteria based on serum creatinine.	69.3% of patients with SAP; 59.2% of patients with SAP required RRT	Mortality was 44.9% in AP with AKI versus 20.5% in AP without AKI
Kumar et al., 2015 [16]	Retrospective analysis of 72 patients with SAP admitted to a tertiary center	AKI was defined and classified according to the RIFLE criteria	19.4% of patients with SAP; 13.9% of patients with SAP required RRT	Mortality was 57% in AP with AKI versus 0 in AP without AKI
Párniczky et al., 2016 [17]	Prospective multicenter study of 600 patients with AP (61% MAP, 30% MSAP, 9% SAP)	Renal failure as an organ complication in patients with SAP; no strict definition given	36% of patients with SAP	Mortality was 43.8% in SAP with renal failure versus 21.4% in SAP without renal failure
Gougol et al., 2017 [14]	Prospective observation of 500 AP patients admitted to a tertiary center	Isolated renal failure according to the modified Marshall scoring system	Isolated renal failure reported in 15% of patients with SAP	No deaths in isolated renal failure versus 22.4% mortality in MOF
Devani et al., 2018 [18]	3,466,493 patients hospitalized with AP (ICD-9 code) between 2003–2012, from Nationwide Inpatient Sample database	AKI was identified using the ICD-9 codes for AKI (584; 584.5; 584.6; 584.7; 584.8; 584.9)	Prevalence of AKI in AP nearly tripled from 4.1% in 2003 to 11.7% in 2012. Overall prevalence within the study period was 7.9%.	Mortality of patients with AKI complicating AP decreased from 17.4% in 2003 to 6.4% in 2012.
Chai et al., 2018 [13]	Retrospective analysis of 237 patients with AP (79% MAP, 16% MSAP, 5% SAP)	2012 KDIGO criteria, any stage	7.6% of all patients with AP	50% of patients with AKI had stage 1 AKI according to 2012 KDIGO criteria
Manokaran et al., 2018 [19]	100 patients with SAP from tertiary hospital	KDIGO 2012, any stage	32% of patients with SAP	Mortality 12.5% in SAP with AKI versus 1.5% in SAP without AKI

Abbreviations: SAP, severe acute pancreatitis; AKIN, Acute Kidney Injury Network; RIFLE, risk, injury, failure, loss of kidney function, end-stage kidney disease; MOF, multi-organ failure; ICD-9, International Classification of Diseases-9; ICU, intensive care unit; MAP, mild acute pancreatitis; MSAP, moderately-severe acute pancreatitis; KDIGO, Kidney Disease: Improving Global Outcomes; RRT, renal replacement therapy.

**Table 2:** The recent evidence on AKI in AP coming from animal studies.

Reference	Description of the Study and Results
Zhang et al., 2014 [36]	Sprague-Dawley rats with SAP was induced by retrograde infusion of 5% sodium taurocholate into the bile-pancreatic duct were treated with caspase-1/interleukin-1β-converting-enzyme inhibitor. The inhibitor attenuated intrarenal IL-1β and caspase-1 expression, the histopathologic changes in kidneys and increased serum creatinine observed in SAP.
Li et al., 2015 [37]	SAP was induced in Male Sprague-Dawley rats by retrograde injection of 5% sodium deoxycholate into bile-pancreatic duct. Serum creatinine and blood urea nitrogen significantly increased in rats with SAP 12 h after surgery. Histological changes in kidney tissue and injury to renal endothelial cells were most pronounced at 36-48 h post-surgery. These changes were preceded by increase in mRNA and protein expression of matrix metalloproteinase-9 (MMP-9), also in active form, and vasodilator-stimulated phosphoprotein (VASP) at 12-24 h post-surgery.
Wu et al., 2017 [26]	Severe hypertriglyceridemia in ApoC III transgenic mice aggravated kidney injury in the course of AP established by retrograde injection of 0.5% sodium taurocholate to pancreatic duct. ApoC III transgenic mice developed more severe pancreatic damage and more advanced histological changes in the kidneys associated with higher serum creatinine than wild type mice.
Kong et al., 2018 [28]	Sprague-Dawley rats with AP induced by retrograde infusion of body weight of 3.5% sodium taurocholate solution into the biliary-pancreatic duct were pretreated with antithrombin III (AT III), or AT III was administered postoperatively. Both ways of AT III administration attenuated increase in serum creatinine, renal tubular detachment, brush border loss, and necrosis of tubular cells.
Gori et al., 2019 [38]	The authors studied the diagnostic utility of urinalysis and urinary gamma glutamyl transpeptidase-to-urinary creatinine (GGT/Cr) in dogs with spontaneously developed AP. Non-survivors showed higher dipstick bilirubin levels and urine protein-to creatinine ratio >2 than survivors. The GGT/Cr was not useful in the prognosis of outcome.
Gori et al., 2019 [39]	The authors studied the prevalence of AKI complicating spontaneously developed AP in 65 dogs. Higher serum urea and creatinine and oligo- or anuria predicted death of the animals. AKI was diagnosed in 26.2% of dogs.

**Table 3:** Laboratory markers evaluated for prognosis or diagnosis of AKI in patients with AP.

Marker	Reference	Study Design and Patients	Definition of AKI	Cut-off Value	Diagnostic Sensitivity	Diagnostic Specificity	AUC
Serum cystatin C	Chai et al., 2018 [13]	Retrospective analysis of 237 patients diagnosed with AP: 5% diagnosed with SAP; 7.6% of all AP patients diagnosed with AKI	KDIGO criteria	1.865 mg/L	88.9%	100%	0.948 (95% CI: 0.875–1.0)
Serum NGAL	Siddappa et al., 2018 [50]	Prospective study of 50 patients with AP admitted to tertiary center: 23 patients diagnosed with SAP, 21 with AKI	Modified Marshall scoring system and AKIN criteria	790.9 ng/mL	64%	96%	0.8
Urine NGAL				221 ng/mL	82%	80%	0.9
Serum procalcitonin	Huang et al., 2013 [64]	305 patients with AP admitted to ICU: 52 cases of AKI	RIFLE criteria	3.30 ng/mL	97.2%	92.3%	0.986 (95% CI: 0.966–1.000)
Serum uromodulin	Kuśnierz-Cabala et al., 2017 [65]	Prospective study of 66 patients with AP: 5 diagnosed with SAP, 11 diagnosed with AKI	KDIGO criteria	no data			0.684 (95% CI: 0.508–0.860)
Serum uromodulin to creatinine ratio							0.846 (95% CI: 0.706–0.987)
Serum angiopoietin-2	Sporek et al., 2016 [66]	Prospective study of 65 patients with AP: 5 diagnosed with SAP, 11 diagnosed with AKI	KDIGO criteria	Higher concentrations of angiopoietin-2 was observed in patients with AKI during first 72 h from the onset of AP. OR for AKI 1.12 (1.02–1.24) at 24 h; 1.37 (1.12–1.68) at 48 h, and 1.49 (1.17–1.90) at 72 h per 1 ng/mL increase in angiopoietin-2			
Serum soluble fms-like tyrosine kinase-1 (sFlt-1)	Dumnicka et al., 2016 [67]	Prospective study of 65 patients with AP: 5 diagnosed with SAP, 11 diagnosed with AKI	Modified Marshall scoring system	OR for renal failure at 24 h from the onset of AP symptoms 1.31 (1.06–1.63) per 10 pg/mL increase in sFlt-1			

Abbreviations: AUC, area under the receiver operating characteristic curve; OR, odds ratio; CI, confidence interval; NGAL, neutrophil gelatinase-associated lipocalin.

**Table 4:** Novel laboratory markers of renal dysfunction or injury associated with prognosis or diagnosis of AP severity.

Marker	Reference	Study Design and Patients	Severity Assessment	Cut-off Value	Diagnostic Sensitivity	Diagnostic Specificity	AUC (95% CI)
Serum NGAL	Sporek et al., 2016 [52]	Prospective observation of 65 adult patients admitted with AP; NGAL was measured at 24, 48 and 72 h from the onset of AP	Moderately severe and severe AP according to 2012 Atlanta Classification versus mild AP	165 μg/L (at 24 h)	63%	80%	0.727 (0.582–0.872)
				183 μg/L (at 48 h)	90%	72%	0.860 (0.773–0.948)
				182 μg/L (at 72 h)	84%	78%	0.843 (0.730–0.956)
Urine NGAL	Lipinski et al., 2015 [53]	Observational cohort study of 104 patients with acute pancreatitis	SAP according to 2012 Atlanta Classification; organ failure according to modified Marshall scoring system	Prediction of SAP: 68.9 ng/mL	81.2%	71.5%	0.750 (0.622–0.890)
				Prediction of MOF: 86.5 ng/mL	75%	76%	0.870 (0.779–0.964)
				Prediction of death: 86.5 ng/mL	75%	74%	0.800 (0.632–0.968)
